# Insights into the beneficial effect of caloric/ dietary restriction for a healthy and prolonged life

**DOI:** 10.3389/fphys.2012.00318

**Published:** 2012-08-09

**Authors:** Rani Pallavi, Marco Giorgio, Pier G. Pelicci

**Affiliations:** European Institute of OncologyMilan, Italy

**Keywords:** caloric restriction, dietary restriction, cancer, anti-tumor effect, aging

## Abstract

Over the last several years, new evidence has kept pouring in about the remarkable effect of caloric restriction (CR) on the conspicuous bedfellows- aging and cancer. Through the use of various animal models, it is now well established that by reducing calorie intake one can not only increase life span but, also, lower the risk of various age related diseases such as cancer. Cancer cells are believed to be more dependent on glycolysis for their energy requirements than normal cells and, therefore, can be easily targeted by alteration in the energy-metabolic pathways, a hallmark of CR. Apart from inhibiting the growth of transplantable tumors, CR has been also shown to inhibit the development of spontaneous, radiation, and chemically induced tumors. The question regarding the potentiality of the anti-tumor effect of CR in humans has been in part answered by the resistance of a cohort of women, who had suffered from anorexia in their early life, to breast cancer. However, human research on the beneficial effect of CR is still at an early stage and needs further validation. Though the complete mechanism of the anti-tumor effect of CR is far from clear, the plausible involvement of nutrient sensing pathways or IGF-1 pathways proposed for its anti-aging action cannot be overruled. In fact, cancer cell lines, mutant for proteins involved in IGF-1 pathways, failed to respond to CR. In addition, CR decreases the levels of many growth factors, anabolic hormones, inflammatory cytokines, and oxidative markers that are deregulated in several cancers. In this review, we discuss the anti-tumor effect of CR, describing experiments done *in vitro* in tumor models and *in vivo* in mouse models in which the tumor was induced by means of radiation or chemical exposure, expressing oncogenes or deleting tumor suppression genes. We also discuss the proposed mechanisms of CR anti-tumor action. Lastly, we argue the necessity of gene expression studies in cancerous versus normal cells upon CR.

## Introduction

Growing awareness that diet and environmental factors have a profound effect in the initiation, promotion, and progression of cancer argues that cancer is a preventable disease. In fact, alteration in the eating habit from traditional to westernized diets appears to correlate with the increased risk of many common cancers in both developed and developing countries (Baade et al., 2009; Kolonel et al., 2004). For example, prostate cancer, which was previously prevalent only in developed countries, showed an increased incidence rate in less developed and developing countries, due to westernization of food habits (Baade et al., 2009). Further, studies of ethnic and migrant groups in Hawaii showed adaptation of Japanese immigrant cancer risk incidences to those of native Hawaiian people (Kolonel et al., 2004). These observations reinforce the belief that environment and diet have an impact on cancer development. The increased risk of breast cancer in Japanese women who migrated to US also supports the influence of environment and diet on the pathogenesis of cancer (Probst-Hensch et al., 2000). In addition, observational studies showing a decreased risk of cancer in a population with a dietary habit enriched for plant food, and limited consumption of animal fat and dairy products, also point toward the importance of diet in cancer (Kushi et al., 2006). Obesity due to over eating has been shown to be associated with increased risk of colon, breast (in post-menopausal women), endometrium, kidney, esophagus, pancreas, prostate, gallbladder, and liver cancer (Calle and Kaaks, 2004). It is believed that increased food consumption can influence the expression of genes involved in important cellular functions, such as DNA repair, cell proliferation and differentiation, and apoptosis, by altering the levels of metabolic hormones and growth factors, and can lead to accumulation of damage and mutations and ultimately malignant transformation (Hursting et al., 1999; Calle and Kaaks, 2004). Therefore, it can be assumed that by controlling our diet we might also control cancer risk. Caloric restriction (CR), which can be defined as “under nutrition without malnutrition,” has emerged as a robust method to decrease cancer incidence, besides increasing the life span of the individuals (Sell, 2003). CR has been shown to reduce the levels of many cancer-causing agents like anabolic hormones, growth factors, and reactive oxygen species (ROS) in animal models. Unfortunately, the exact metabolic adaptation through which CR exhibits its anti-tumor effect is not completely understood. However, at least in part, the mechanism responsible for the anti-tumor effect of CR involves a similar metabolic adaptation as seen in the case of its anti-aging effect. The anti-proliferative and pro-apoptotic properties of CR, in addition to its ability to decrease oxidative stress and maintain genomic stability, could be responsible for its anti-tumor activity.

Here, we discuss the existing evidence regarding the anti-tumor activity of dietary/energy restriction and factors and pathways crucial for its effect. We also discuss the putative parallel mechanisms through which CR exerts both anti-aging and anti-tumor activities. An insight into how anti-aging effects may also lead to tumorigenesis is also provided. Finally, we discuss the potential of CR interventions at clinical level.

## Caloric restriction as a promising natural approach to overcome cancer

**Figure 1:**
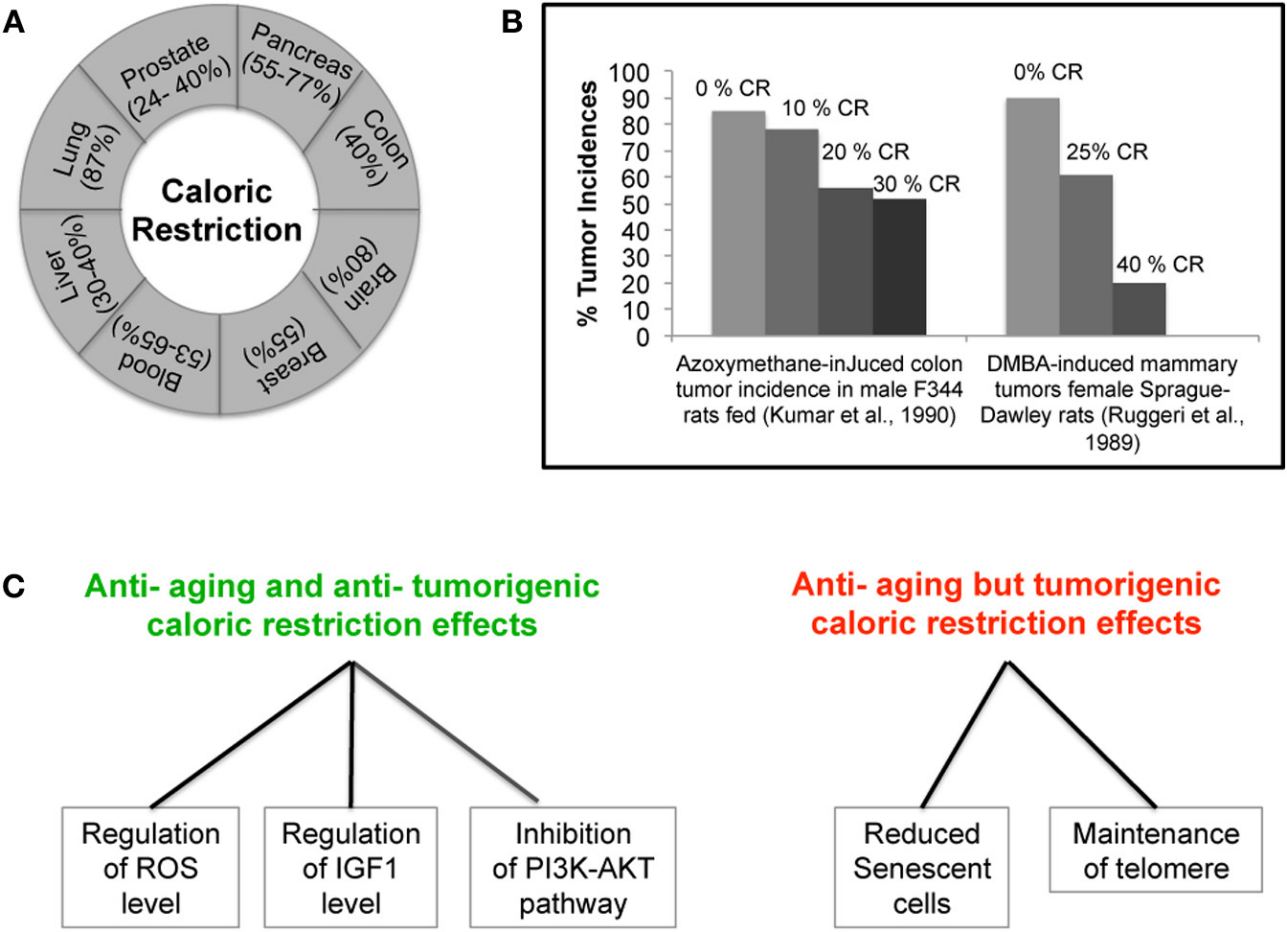
**Caloric Restriction (CR) and Cancer. (A)** CR effectively inhibits various kinds of cancer in animal models. **(B)** Correlation between decrease in the incidence of tumor and severity of CR. **(C)** Demonstration of parallel and opposing effects of CR on cancer and aging. The ability of CR to decrease the levels of IGF-1 and ROS and inhibit the PI3K-AKT pathway can simultaneously protect cells from aging and cancer. However, CR ability to maintain telomere length and reduce the frequency of senescence can promote cancer but may be also beneficial in delaying aging.

Realization that both the environment and the diet of an individual can influence the occurrence of cancer has heightened the idea that cancer is a preventable disease. Epidemiological studies have also shown a correlation between weight of an individual and development of tumors suggesting that controlling the diet may contribute to cancer prevention (Tannenbaum, 1940, 1942; Collaborative Group on Epidemiological Studies of Ovarian Cancer, 2012). In fact, beside the recognized effects that restriction in calorie intake has on aging, increasing evidence also supports a role of CR in inhibiting tumor. The first ever observation on the anti-tumor activity of CR was made in the early 1900's. One of the very first experiments in mice and rats showed that lowering the weight by CR can lower the frequency of various types of spontaneous tumors and other inducible tumors (Mccay et al., 1939; Tannenbaum, 1940). More recently, experiments carried out in tumor susceptible C3H/He female mice showed that a change in diet to 70% of the *ad-libitum* diet could single-handedly suppress the spontaneously occurring mammary tumor, suggesting a new way of restricting tumor growth (Kharazi et al., 1994). The fact that CR is linked to a reduction of the levels of mouse mammary tumor virus (MMTV) RNA and incidences of spontaneous mammary tumor further reinforces this belief (Li et al., 1994). Furthermore, in an attempt to find out differences in tumor biology with age, Pili et al. found that young mice that were otherwise vulnerable to tumor growth and expansion as compared to old mice, when fed on a caloric restricted diet showed a decrease in the growth of transplantable tumors and decreased angiogenesis (Pili et al., 1994). In addition, CR has also been shown to be effective against chemically and radiation induced leukemia, and mammary and liver tumors (Beth et al., 1987; Ruggeri et al., 1989; Fu et al., 1994; Yoshida et al., 1997, 1999, 2006). The anti-tumor ability of CR was also observed in animal models of pancreatic, colon, breast, prostate, and lung tumor, proving it to be active against various kinds of cancer (Figure 1A) (Bunk et al., 1992; Roebuck et al., 1993; Mukherjee et al., 1999, 2002; Dirx et al., 2003a,b; Mai et al., 2003; Phoenix et al., 2010; Lashinger et al., 2011). The inhibitory effect of CR appears to depend on a caloric intake restriction ranging from 25% up to 60% of *ad-libitum* levels, combined with adequate intakes of essential nutrients. Interestingly, experiments in rats showed that by increasing the degree of CR intervention, the reduction in chemically-induced tumor incidence was intensified (Figure 1B) (Ruggeri et al., 1989; Kumar et al., 1990). Most importantly, a very recent study, using a mouse model of post-menopausal obesity, provided the evidence that CR can break the obesity-cancer progression link offering a new hope to women vulnerable to post-menopausal breast cancer (Nogueira et al., 2012). Several studies using mouse models of brain tumor showed CR to be effective not only in non-invasive tumors but also in the most aggressive and invasive forms of brain tumor, proving it to have anti-proliferative, anti-angiogenic, and anti-invasive properties (Mukherjee et al., 2002, 2004; Zhou et al., 2007; Shelton et al., 2010). Recently, CR has also been shown to be beneficial for mice lacking the tumor suppressor p53. In fact, mice lacking p53 develop lymphoma by six months of age and die very early; however, when put on a calorie restriction diet, these mice live longer due to decreased tumor incidence (Hursting et al., 1994, 1997). This is very interesting as p53 is known to be non-functional in almost all types of human cancer, either because of its own mutation or mutation in its regulator/s. This last observation again indicates that CR might constitute an effective intervention for a prolonged healthier life. So far, the only tumor types which failed to respond to calorie restricted diets are the tumors carrying a mutation in either PI3K or PTEN genes, thus leading to the constitutive activation of the PI3K pathway (Kalaany and Sabatini, 2009). Table 1 summarizes the amount of calorie restriction in percentage or kcal/day or kcal/week in the above mentioned studies.

**Table 1:** **Summary of the extent of caloric restriction and its effect on tumor growth**.

**Mice/Rat strain**	**Control intake (type or kcal/day)**	**Calorie restriction**		**Outcome**	**Reference**
		**Amount in kcal/day**	**% Restriction with respect to *ad-libitum* diet**		
Female sprague dawley rats	50 kcal/day	35 kcal/day	30	Decreased MNU induced carcinoma	Bunk et al., 1992
Sukling male lewis rats	84 kcal/day	~75.6 kcal/day, ~71.5 kcal/day, ~67.3 kcal/day, ~58.9 kcal/day	10, 15, 20, 30	CR inhibited Azaserine-induced preneoplastic lesion The inhibition increased with more severe restriction regimens	Roebuck et al., 1993
Balb/c	*Ad-libitum* (standard chow diet) or high energy diet	-	30	CR reduced tumor growth and metastasis in aggressive model of hormone independent breast cancer in syngeneic model using triple negative 66cl4 tumor cells in Balb/c mice	Phoenix et al., 2010
Apc (Min) mouse	*Ad-libitum* AIN-76A diet	-	40	Intestinal polyp was reduced by 57% by CR	Mai et al., 2003
Male FischerX cophenhagen F1 rats	*Ad-libitum* AIN-76A diet (61–69 kcal/day)	~44.53 kcal/day	(1) 30% total diet restriction (2) 30% carbohydrate restriction (3) 30% lipid restriction	Each of the 3 different restriction diets inhibited R3327 tumor to the same extent	Mukherjee et al., 1999
Adult male SCID mice	*Ad-libitum* AIN-76A diet (15.4 kcal/day)	11.1 kcal/day	(1) 30% total diet restriction (2) 30% carbohydrate restriction (3) 30% lipid restriction	Each of the 3 different restriction diets inhibited LNGP human carcinoma to the same extent	Mukherjee et al., 1999
C57BL/6J and BALBc/J-SCID	*Ad-libitum* PROLAB Chow diet (18 to up to 24 kcal/day)	13 kcal/day	30%	CR reduced intra- cerebral CT-2A tumor growth and angiogenesis in syngeneic CT-2A experimental mouse brain tumor	Mukherjee et al., 2002
C57BL/6J and BALBc/J-SCID	*Ad-libitum* PROLAB Chow diet (12–14 to up to 20–24 kcal/day)	-	40%	CR decreased vascularity (factor VIII) and increased apoptosis in three distinct models of brain tumor (a) A malignant mouse astrocytoma (CT-2A) (b) A human glioma (U87-MG) (c) Mouse ependymoblastoma	Mukherjee et al., 2004
C57BL/6	*Ad-libitum*	-	30%	CR inhibited MMTV-Wnt1 tumor growth in mouse model of post-menopausal obesity	Nogueira et al., 2012
VM/DK (VM)	*Ad-libitum*	-	60%	CR effectively reduce malignant brain tumor growth in VM-M3 GBM model	Shelton et al., 2010

## Proposed mechanisms for the anti-tumor activity of caloric restriction

The mechanisms responsible for the observed effect of CR in aging, cancer and other chronic diseases are still under scrutiny. The initial studies indicated the involvement of similar mediators and pathways for both the anti-aging and anti-tumor activity of CR. Accumulation of cellular damage is assumed as one of the initiating events in aging and cancer. ROS, which are natural byproducts of cell normal metabolism and are capable of damaging macromolecular components of the cells, including proteins, lipids and DNA, are considered as the main culprit. In the cells, ROS are neutralized by the action of anti-oxidant enzymes to avoid oxidative damage. However, as the cells age, their capacity to neutralize ROS diminishes, leading to accumulation of macromolecular damage. Moreover, tumorigenesis is also fuelled by accumulation of cellular damage, partly by increased intrinsic ROS stress due to oncogene stimulation, increased metabolic activities, and mitochondrial malfunction (Pelicano et al., 2004). Thus, regulation of oxidative stress could be the mechanism in common between CR anti-tumor and anti-aging activities. In fact, 25–40% CR was shown to maintain the otherwise diminishing levels of anti-oxidant defense systems in aging rodents (Youngman et al., 1992). Further, 30% CR was shown to decrease nitric oxide production in p53 deficient mice and delay tumorigenesis (Hursting et al., 1994, 2001; Mei et al., 1998). The ability of CR to reduce oxidative stress was recently shown to be dependent on SIRT3, a deacetylase (Qiu et al., 2010) and mice deficient in SIRT3 were shown to be prone to both aging and cancer and failed to respond to CR (Kim et al., 2010).

Another highly studied common mediator for the anti-tumor and anti-aging activity of CR is IGF-1 (Insulin Growth Factor 1). The involvement of IGF-1 in aging is supported by the availability of nutrition studies in yeast, nematode, fruit fly and mouse (Gems and Partridge, 2001). Mutants of *Drosophila* that exhibited alteration of insulin/IGF-1 signaling pathways lived longer (Clancy et al., 2001; Tatar et al., 2001). Life span extension in *C. elegans* requires deregulation of Insulin/IGF-1 signaling (Lin et al., 2001). The importance of IGF-1 in life span extension is also observed in genetically modified mice defective for growth hormone (GH) or IGF-1 production (Flurkey et al., 2001). These mice live longer as compared to wild type mice. The production of IGF-1 by the liver was shown to be stimulated by GH (Isaksson et al., 1987). Many of the several biological roles of GH seem to depend on its interaction with the growth hormone receptor/binding protein (GHR/BP). Coschigano and colleagues reported that mice with disruption in GHR/BP, although showing high levels of circulating GH, had lower serum levels of IGF-1 in comparison to wild type, and lived longer (Coschigano et al., 2000). All these studies show the importance of the IGF-1 pathway in aging. CR was invariably shown to decrease the IGF-1 serum levels in animal studies (Weindruch and Walford, 1988; Ruggeri et al., 1989; Hursting et al., 1993; Berrigan et al., 2002). Interestingly, the injection of IGF-1 in the mice reversed the CR effect. Furthermore, the ability of CR to regulate IGF-1 levels and the Insulin/IGF-1 pathway could also be responsible for its anti-tumor effect as an elevated IGF-1 serum level is associated with an increased risk of breast, prostate, colon, and lung cancer in humans (Chan et al., 1998; Hankinson et al., 1998; Schaefer et al., 1998; Wolk et al., 1998; Ma et al., 1999; Yu et al., 1999). Involvement of IGF-1 in cancer is further supported by its ability to enhance the growth of a variety of cancer cell lines (Macaulay, 1992; LeRoith et al., 1995; Singh et al., 1996). The tumorigenic property of IGF-1 is thought to be due to its ability to regulate mitogenic and anti-apoptotic pathways (Resnicoff et al., 1995; Yu and Rohan, 2000). In fact, in a transplantable and spontaneous leukemia mouse model, CR has been shown to reduce serum IGF-1 level and decrease leukemia cell proliferation (Hursting et al., 1993). Interestingly, the anti-proliferative effect of CR on leukemia cells was annulled by restoration of serum IGF-1 concentration (Hursting et al., 1993). Similarly, in p53 deficient mice, restoration of IGF-1 levels reverses the beneficial effect of CR on p-cresidine induced carcinogenesis (Dunn et al., 1997). Hence, it can be assumed that, at least in part, CR modulation of IGF-1 mediates its anti-tumor and anti-aging effects.

Recently, the Forkhead box-O (FOXO) family of proteins has been shown to be required for the anti-tumor and anti-aging activity of CR (Greer et al., 2009; Yamaza et al., 2010). The FOXO family of transcription factors is a direct target of the PI3K-AKT pathway (Lin et al., 1997; Ogg et al., 1997). Activation of the PI3K-AKT pathway leads to the phosphorylation and inactivation of FOXO by AKT. However, its phosphorylation by AMPK enhances its transcriptional activity (Greer et al., 2009). FOXO transcription factors, by modulating specific targets genes (p21, p27, cyclin G2, BIM1, Bcl-6, FasL, GADD45, MnSOD, catalase), promote a variety of cellular responses such as cell cycle arrest, apoptosis, DNA repair and resistance to cellular stress (Greer and Brunet, 2005), hence representing an attractive tumor suppressor candidate. Interestingly, FOXO3 has been found to be deregulated in breast cancer (Hu et al., 2004). Moreover, expression of an active form of FOXO suppresses tumor in transplanted nude mice (Hu et al., 2004; Yang et al., 2005). Further, expression of a constitutive active form of FOXO has been shown to inhibit tumorigenesis in PTEN-null cells (Ramaswamy et al., 2002). An indication of the involvement of FOXO in longevity comes from a study in mutant worms where the increased longevity, due to mutations in the insulin receptor and PI3K, was reversed by an additional mutation in the FOXO ortholog Daf-16 (Lin et al., 1997; Ogg et al., 1997; Kenyon, 2005). Studies in worm have shown that CR ability to extend life span is dependent on AMPK, which partly acts via FOXO (Greer et al., 2007). The inability of CR to extend life span in the FOXO (daf-16) mutant strongly indicates FOXO as a mediator of its anti-aging activity (Greer et al., 2007). The requirement of FOXO in the antineoplastic effect of CR has been shown using FOXO1 knockout heterozygous mice. In comparison to wild type mice, these mice failed to recapitulate the beneficial effect of CR on tumor development. Wild type mice on CR diet showed lower incidence of tumor and tumor related deaths as compared to FOXO1 knockout heterozygous mice (Yamaza et al., 2010), thus suggesting FOXO as a mediator of CR anti-tumor effects as well.

## The divergent mechanism of caloric restriction in aging and cancer

The assumption that the anti-tumor ability of CR is a parallel effect of its anti-aging activity and *vice-versa* is a debatable issue. The regulation of GHs, oxidative stress, DNA damage, and metabolic pathways by CR could simultaneously result in its anti-tumor and anti-aging activities (Figure 1C). Recent studies have shown that CR reduces the frequency of senescent cells in the liver and small intestine of mice (Wang et al., 2010). CR effect on cellular senescence, a cause of aging, could be crucial to its anti-aging activity (Goldstein, 1990; Wang et al., 2010). However, this property of CR could also be beneficial to tumor cells where induction of senescence is an effective tumor suppressor mechanism (Lleonart et al., 2009). Further, CR has been shown to maintain telomere length, another process important for tumorigenesis (Feldser and Greider, 2007; Wang et al., 2010). Experiment by Oliverras-Ferraros showed that cancer cell lines can be maintained in culture for several months in the presence of CR mimetics (CRM) (Oliveras-Ferraros et al., 2010). Gene expression analysis suggests the retrogression from a more differentiated state to a stem like primitive step in the presence of CRM (Oliveras-Ferraros et al., 2010). While this finding promises potential applications in the replacement of adult aging tissues, it is, at the same time, a matter of concern as regards tumor biology. Another interesting observation is the regulation of SIRT1 by p53 in response to CR. It seems that the induction of SIRT1 under the condition of nutrient deprivation requires occupancy of its promoter by p53. Any mutation that affects the binding of p53 to the SIRT1 promoter region affects SIRT1 up-regulation in response to CR. Therefore, it appears that the SIRT1-mediated beneficial effect of CR requires an active p53 and its binding on SIRT1 (Naqvi et al., 2010). However, the ability of CR to exert its ant-tumor effect in the absence of p53, further points towards the singular regulation of aging and cancer by CR (Hursting et al., 1997). In this context, it is essential to understand how CR carries out both anti-tumor and anti-aging activities, and whether its effects occurs through the same mechanism but with parallel and opposing results on cancer and aging. Further investigations are required (Figure 1C).

## Other possible mechanisms for the anti-tumor activity of caloric restriction

While one of the mechanisms responsible for CR-mediated beneficial effects on cancer has been shown to involve the same metabolic adaptation implicated in its anti-aging effects, the role of other specific mediators and pathways cannot be ruled out. One possibility could be the regulation of oncogenes and tumor suppressor genes by CR. For example, gene expression analysis of liver from mice fed on caloric restricted diet revealed significant changes in the genes involved in p53 dependent cell cycle and apoptosis (Estep et al., 2009). One of the most highly up regulated genes in the liver of CR fed mice was DNA-damage inducible transcript 4 (Ddit4), a p53 controlled negative regulator of the m-TOR pathway (Wei et al., 2006; Estep et al., 2009). Ddit4 is a known tumor suppressor whose expression has shown to be down regulated in a subset of human cancers (Deyoung et al., 2008). CR mediated up-regulation of the Ddit4 transcript could be one of many ways by which CR exerts its anti-tumor effect. Analysis of pancreatic acinar cells from CR-fed Brown Norway Rat revealed reduced expression of the c-Ha-Ras oncogene and reduced mutations in the p53 gene (Hass et al., 1993). Further, study in mouse mammary tumor/v-Ha-ras transgenic mice showed that a restricted diet decreases the tumor incidence in these mice, may be through CR mediated increased levels of the tumor suppressor p53 and scavenging enzymes and decreased levels of c-erbB2 and v-Ha-ras RNA (Fernandes et al., 1995). p27/kip is a cyclin dependent kinase inhibitor whose activity is deregulated in various kinds of cancer (Slingerland and Pagano, 2000; Bloom and Pagano, 2003). It has been proposed that CR inhibits induced mammary carcinogenesis by arresting cell cycle progression *via* up-regulation of the expression of p27/kip (Zhu et al., 1999). These observations further point towards the ability of CR to modulate the expression of oncogenes and tumor suppressor genes. Recently, epigenetic regulation by CR has been proposed as one of many mechanisms through which CR controls aging (Li et al., 2011). In fact, it has been shown that CR, by modulating epigenetic changes such as DNA methylation or histone modification, controls the expression of oncogenes and tumor suppressors (Hass et al., 1993; Li et al., 2010). The ability of CR to hyper-methylate the promoter of proto-oncogenes such as Ras, thus leading to their silencing, could contribute toward cancer prevention (Hass et al., 1993).

A recent study using WI-38 (normal cells) and SV-antigen transfected immortalized WI-38 cells (precancerous cells) showed that glucose restriction displayed an altered regulation of the expression of both hTERT and the tumor suppressor p16 in normal and precancerous cells. In normal cells, glucose restriction leads to increased expression of hTERT and decreased expression of p16, leading to delayed aging. However, in precancerous cells, glucose restriction leads to decreased expression of hTERT and increased expression of p16 leading to apoptosis (Li et al., 2010), suggesting that CR may mediate its anti-aging and anti-tumor activities *via* differential regulation of oncogenes and tumor suppressors by varied chromatin modifications. It can be also assumed that, through chromatin modulation, CR might bring out differential gene expression in normal and cancerous cells. Therefore, it would be interesting to examine the effect of CR on the gene expression profile of normal and cancerous cells.

## Clinical implications of caloric restriction

Although, reduction in calorie intake has emerged as a most potent broadly acting intervention that prevents cancer in experimental animals, its role at clinical level is yet to be defined. There are limited numbers of designed and controlled studies that are aimed to find out the efficacy of CR in humans. This is mainly because of the unavailability of human volunteers willing to follow a restricted diet regime despite CR claimed ability to provide a healthy prolonged life. However, epidemiological studies and observations from both natural and historical situations have indicated CR to be effective in humans too. Many of these analyses were carried out from the answers to questionnaires filled by human volunteers regarding their history, such as a study involving Spanish nursing residents indicating a beneficial effect of CR (Roth et al., 1999). Interestingly, studies utilizing data from cancer registries have shown a correlation between weight loss during adulthood and occurrence of breast cancer. Women's cohorts who experienced a weight loss in adulthood had reduced risk of developing breast cancer in comparison to the ones who gained weight (Trentham-Dietz et al., 2000; Harvie et al., 2005; Christou et al., 2008; Kawai et al., 2010). Likewise, a retrospective study in Swedish women who suffered from severe anorexia nervosa showed that they had a 53% lower incidence of breast cancer than the Swedish general population (Michels and Ekbom, 2004). Similarly, a decreased incidence of breast cancer was observed in Danish women suffering from anorexia nervosa, and in Norwegian pre-pubertal girls and Dutch women who had been exposed to famine during World War II (van Noord and Kaaks, 1991; Tretli and Gaard, 1996; Mellemkjær et al., 2001). Further, the women of the Okinawa community, who follow a traditional lower calorie diet, have lower incidence of breast cancer compared to other Japanese women (Willcox et al., 2007). These observations indicated that starvation or CR during adolescence and adulthood had clear impact on the development of breast cancer, as observed in the rodents. Most interestingly, the decreased prevalence of cancer and vascular diseases in the Okinawa community, due to less calorie intake habit, is considered to be responsible for their lower mortality rate and for their tumor free longer life (Kagawa, 1978). The questionnaire based study of the Netherlands Cohort, who experienced severe CR as adolescent during the Hunger Winter of World War II, has shown that energy restriction during childhood and adolescence also decreases the risk of colorectal and ovarian cancer (Dirx et al., 2003a,b; Hughes et al., 2009; Schouten et al., 2011). All these studies point toward a role for CR in the modulation of human cancer development.

Apart from these retrospective and historic observations, controlled studies involving cancer patients also indicated a promising effect of CR on cancer. A study involving the enrollment of obese persons in a weight loss program based on CR, showed a reduction in their rectal cell proliferation, a biomarker for colon carcinogenesis, suggesting that CR may prevent colon cancer (Steinbach et al., 1994). Another case report using a ketogenic diet that resulted in low blood glucose levels, as seen in caloric restricted animals, showed a decrease in tumor metabolism (Nebeling et al., 1995). These observations are encouraging and suggestive of the clinical potential of CR and merit further research.

## Future directions

Considering the robustness of the data regarding the beneficial effect of CR on a diverse range of ailments, further scrutiny of CR methods/application must be sought. As extensive CR is impractical to achieve in humans, studies directed at understanding the mechanism of action of CR are essential. These kinds of studies are required for the identification of effectors or pathways that could possibly be targeted to achieve the beneficial effect of CR. As discussed above, CR might exert its effect on various human ailments through different mechanisms. However, no direct evidence is available. Therefore, the mechanism of action of CR should not be assumed universal and needs to be examined in each disease condition.

The effect of CR on energy balance should not be ignored. There is growing evidence to suggest the association of energy balance including diet, weight, adiposity, and physical activity with tumorigenesis. It has been shown that increased energy expenditure due to increasing physical activities can reduce obesity and might be beneficial in delaying tumorigenesis, at least in some of the mouse models of mammary tumor (Cohen et al., 1988; Thompson et al., 1995; Thompson, 1997; Jakicic and Otto, 2005). However, as results on the tumor inhibiting ability of energy expenditure by exercise are controversial, a more extensive examination is required (Cohen et al., 1992; Gillette et al., 1997; Thompson et al., 1988). Additionally, a detailed and systematic quantitative analysis of the effect of energy intake, energy expenditure and energy balance on tumorigenesis and aging is warranted for the further scrutiny of this process.

One emerging alternative to CR is the use of CRMs. One of the known CRMs, Rapamycin has been shown to be effective in delaying aging as well as tumor growth, two main features of CR benefit. However, this field is still very undeveloped and requires more attention. Of course, novel CRMs might be discovered in the course of dissecting CR mechanisms.

**Figure 2:**
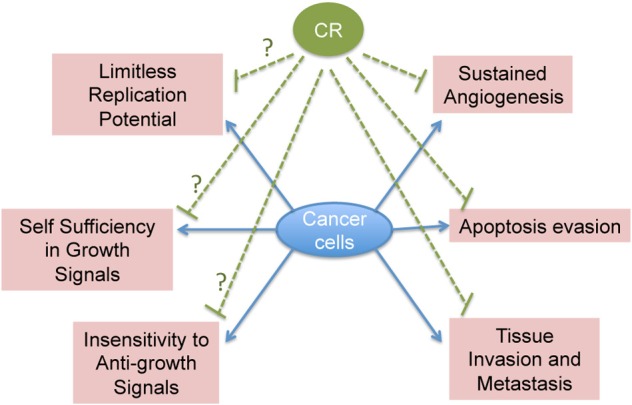
**Schematic representation of CR known and potential effects on cancer hallmarks**.

Another unexplored area of research is the effect of CR on tumor suppressor and tumor promoter genes. Does CR exert its anti-tumorigenic effect through up-regulation of tumor suppressor genes or down-regulation of oncogenes? Interestingly, the p53 family of tumor suppressors has been shown to negatively regulate the Insulin-like Growth Factor 1 (IGF-1) Receptor (IGFR-1), an important player of the insulin receptor pathway, through which CR has been proposed to exert its action (Bruchim et al., 2009). Though, there is evidence that CR can increase apoptosis in tumor cells and can inhibit angiogenesis and invasive properties of cancer cells, its effect on other hallmarks of cancer is not explored (Mukherjee et al., 2002, 2004, 2008; Zhou et al., 2007; Shelton et al., 2010). Therefore, it would be also interesting to examine the ability of CR to target all the hallmarks of cancer (Figure 2).

Another very exciting and unexplored area is that of stem cell biology and CR. Although, a limited number of studies point toward the inhibitory effect of CR on progenitor stem cell proliferation, these need further validation (Yoshida et al., 1997). Finally, studies using human subjects are foremost important for the validation of CR efficacy. In all, we can say that CR is the only natural approach emerging as a conqueror against aging and cancer, and may pave our way toward a healthy prolonged life.

### Conflict of interest statement

The authors declare that the research was conducted in the absence of any commercial or financial relationships that could be construed as a potential conflict of interest.
